# Beyond the core microbiome: endemic bacteria drive functional and microdiversity differences across salamander populations

**DOI:** 10.1093/ismeco/ycag106

**Published:** 2026-04-17

**Authors:** Ostaizka Aizpurua, Elsa Brenner, Garazi Martin-Bideguren, Ion Garin-Barrio, Carlos Cabido, Antton Alberdi

**Affiliations:** Center for Evolutionary Hologenomics, Globe Institute, University of Copenhagen, Øster Voldgade 5-7, 1350 Copenhagen, Denmark; Center for Evolutionary Hologenomics, Globe Institute, University of Copenhagen, Øster Voldgade 5-7, 1350 Copenhagen, Denmark; Center for Evolutionary Hologenomics, Globe Institute, University of Copenhagen, Øster Voldgade 5-7, 1350 Copenhagen, Denmark; Department of Herpetology, Aranzadi Science Society, Zorroagagaina 11, 20014 Donostia-San Sebastian, Spain; Department of Herpetology, Aranzadi Science Society, Zorroagagaina 11, 20014 Donostia-San Sebastian, Spain; Center for Evolutionary Hologenomics, Globe Institute, University of Copenhagen, Øster Voldgade 5-7, 1350 Copenhagen, Denmark

**Keywords:** amphibian, Calotriton, MAG, metagenomics, newt, microbiome

## Abstract

Population-specific variation in animal microbiomes is well documented, yet the functional consequences and underlying mechanisms remain poorly understood. To address this, we conducted genome-resolved metagenomic analyses on gut and skin microbiomes from four populations of Pyrenean brook salamanders (*Calotriton asper*) inhabiting two distinct environments: Pyrenean subalpine brooks and Atlantic montane streams. From paired faecal and skin swab samples, we reconstructed 539 and 43 metagenome-assembled genomes, respectively, and examined taxonomic composition, metabolic capacity, and microdiversity across environments. Although alpha diversity remained similar across environments, both gut and skin microbiomes exhibited significant differences in community composition and functional potential between environments. Partitioning the gut microbiome into core, endemic, and marginal fractions revealed a dominant core community—shared across environments and accounting for over 85% of reads—that did not drive functional divergence. Instead, functional differences were primarily shaped by low-abundance, population-specific endemic bacteria. Atlantic salamanders hosted endemic taxa with significantly greater metabolic potential and higher strain-level microdiversity than those at the Pyrenees. These patterns were not associated with broad-scale dietary differences and may reflect environmental influences such as temperature and nutrient availability. Our findings highlight the relevance of rare, endemic bacteria in driving microbiome function and underscore the power of genome-resolved metagenomics to uncover functional and evolutionary dynamics in wild host–microbe systems.

## Introduction

Population-specific microbiome variation is a well-documented pattern observed across a wide range of animal hosts [1], including humans [2], other vertebrates [3], and invertebrates [4]. The causes and consequences of this variation have been explored from multiple perspectives, such as industrialization in humans [5], ecotypic differentiation in killer whales [6], and habitat degradation in howler monkeys [7]. In some cases, microbiome variation is limited to taxonomic turnover, where one bacterial species replaces another with similar functional properties, maintaining ecological stability within the host microbiome [8, 9]. Such compositional shifts often have minimal biological impact, as overall microbiome function remains unchanged [10]. In contrast, other microbiome alterations lead to significant functional differences [11], reshaping microbial dynamics and potentially influencing host physiology and health [12]. Despite the recognition of these patterns, the mechanisms driving functional divergence in microbiomes remain largely unexplored. While amphibian research has extensively documented spatial and temporal variation in skin and gut microbiome composition [13–15], the functional consequences of these taxonomic shifts remain comparatively understudied. Moreover, only a handful of studies have used metagenomic approaches to uncover the functional capacities of amphibian microbiomes [16–18].

A useful approach to understand the mechanisms driving geographical microbiota variation is to decompose its structure based on the prevalence of its members [19]. Numerous studies have attempted such classifications, often leading to varying definitions of the “core microbiome” depending on the context, study system, and research objectives [20, 21]. Within a spatial eco-evolutionary framework—where microbiomes are examined in relation to animal hosts across geographic ranges—we propose a classification into three distinct fractions: core, endemic, and marginal microbiota. Core microbiota consists of bacterial taxa consistently associated with the host across all environments and geographic regions. This group may include bacteria vertically transmitted from parents to offspring [22], as well as ubiquitous bacteria repeatedly acquired from the environment through common host behaviours, such as feeding or roosting [23]. In contrast, endemic microbiota consists of bacteria that associate with the host only in specific environments or geographic regions. Their presence may reflect loss of vertical transmission, ecological variation such as environmental or dietary shifts that promote local establishment [24], or host genetic variation that exerts different constraints [25]. Although not widespread across the entire host species, endemic bacteria are prevalent within their respective geographic areas, suggesting ecological processes that sustain their persistence. Endemic bacteria may introduce unique functional traits relevant to local host adaptation, such as specialized metabolic capabilities [26]. Lastly, marginal microbiota include bacteria found in only a few individuals with no consistent patterns. Such microbes may be transient, introduced through diet and environmental interactions, or more persistent but specific to individual hosts rather than population-wide trends.

**Figure 1:**
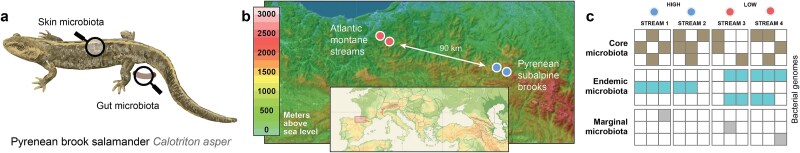
Overview of study design. (a) Representation of the two types of samples collected from Pyrenean brook salamanders (*Calotriton asper*). (b) Geographic location of the four streams on the western edge of the Pyrenees mountain range, classified as Atlantic montane streams and Pyrenean subalpine brooks. (c) Diagram of the theoretical partitioning of the gut microbiota into three fractions: core, endemic, and marginal.

To test whether microorganisms belonging to different fractions harbour different properties, we investigated the gut and skin microbiomes of Pyrenean brook salamanders (*Calotriton asper*) across two distinct environments—Pyrenean subalpine brooks and Atlantic montane streams—spanning four populations (Fig. 1a and b). Using genome-resolved metagenomics, we generated the first comprehensive catalogue of bacterial genomes associated with this species and characterized the functional attributes of its microbiome. We then analysed spatial variation in functional microbiome features across environments and streams. By partitioning the microbiome into core, endemic, and marginal fractions (Fig. 1c), we identified the sources of functional differences, and we employed strain-level analyses to reveal microdiversity differences across environments.

## Materials and methods

This study was conducted within the framework of the Earth Hologenome Initiative (EHI), thus adhering to its standardized methodological procedures for sample collection, preservation, laboratory processing, and bioinformatics [27].

### Study species

The Pyrenean brook salamander (*C. asper*) is endemic to the Pyrenees, the 800 km-long mountain range that separates the Iberian Peninsula from the rest of continental Europe (Fig. 1a). Across its distribution range, populations display distinct genetic diversity with a positive correlation with altitude [28]. Surrounded by peaks exceeding 3400 m above sea level (ASL), these salamanders predominantly inhabit high-elevation (up to 2500 m ASL) streams and lakes with sparse vegetation, where they feed primarily on aquatic invertebrates. While the main population within the Pyrenean mountain range is abundant and well-connected, smaller, peripheral low-elevation populations exist in the surrounding mountains. One such population resides close to the Atlantic coast in the Basque Country, ~90 km from the main population. These isolated peripheral populations inhabit markedly different environments compared to the Pyrenean populations, living in spring-fed streams surrounded by dense Atlantic forests. Given these environmental contrasts, it is likely that the two populations have developed different adaptations to thrive under their respective environmental conditions.

### Field sampling

Field sampling was conducted in four streams in August 2023 in the Northern Iberian Peninsula. The two Pyrenean subalpine brooks, Erlan and Harpea, are situated in different watersheds on the eastern edge of the Pyrenees mountain range at elevations exceeding 1000 m ASL, separated by an approximate linear distance of 3 km. In contrast, the two Atlantic montane streams, Goizueta and Leitzaran, are located ~90 km west of the Pyrenees’ western edge, at elevations <500 m ASL, separated by an approximate linear distance of 4 km (Fig. 1b).

A total of 31 adult male salamanders (Pyrenean subalpine streams = 15 and Atlantic montane streams = 16) were captured with hand-nets, weighed, measured, and kept in individual containers containing stream water until defecation or for up to 2 h. Animal handling was conducted using nitrile gloves, replaced between each animal, ensuring no direct contact between the salamander and human skin. Two types of samples were collected from each captured individual: skin swabs and faecal samples. Skin samples were obtained following the recommended amphibian swabbing protocols described in Hyatt *et al.* [29] and Boyle *et al.* [30] modified for microbial analysis by first rinsing the salamander with sterile water to reduce debris and wash away environmental microbes, and then gently swabbing the dorsal and ventral skin for 30 s each, using single-use sterile FLOQSwabs (Copan Diagnostics, Italy) moistened with sterile water. Faecal samples were collected from the water immediately after defecation. Both faecal samples and skin swabs were stored in standardized EHI collection tubes, containing 1 ml DNA Shield (Zymo, cat. Number R1100-250). Samples were frozen on the day of collection and kept at −20°C until DNA extraction.

### Laboratory sample processing

Samples were mechanically lysed using Lysing Matrix E 96-tube Rack (1.2 ml, MP Biomedicals™, cat. Number 116984010) on a TissueLyser II (Qiagen) for two 6-min cycles at 30 Hz, inverted between TissueLyser runs. In the case of skin samples, swabs were kept inside the tube to maximize cell and DNA elution into the buffer. Subsequently, samples were centrifuged and DNA extraction was conducted from 200 μL of the supernatant using the standardized EHI DNA extraction procedure DREX [31]. Samples were randomized to minimize batch effects, and DNA extraction blanks were included at the beginning of the extraction. These blanks consisted of the same preservation buffer that was used for sample storage and were included throughout all laboratory steps.

DNA sequencing libraries were generated using the Blunt-End Single Tube protocol with BEDC3 adaptors [32]. Libraries were purified by adding 1.67 times the library volume of Solid Phase Reversible Immobilization (SPRI) beads. The mixture was incubated for 5 min at room temperature (ca. 21°C), followed by a double wash with 80% ethanol on a 96S Super Magnet (Alpaca, SKU: A001322). The purified DNA was eluted in Elution Buffer Tween (EBT; Buffer EB, Qiagen, cat. Number 19086, and TWEEN® 20, Sigma–Aldrich, cat. Number P9416-50ML) solution by incubating at 37°C for 10 min. Library preparation performance was assessed by means of a qPCR assay conducted on a Mx3005 qPCR System (Agilent, USA) using 1:20 diluted libraries. qPCR mixes were composed of 2.5 μL 10x PCR Gold Taq buffer, 2.5 μL MgCl_2_ (25 mM), 0.2 μL dNTP mix (10 mM each), 1 μL (10 μM) of IS7 and IS8 primers, 0.5 μL AmpliTaq GOLD DNA polymerase, 1 μL SYBR green dye, 2 μL diluted sample library and 14.3 μL sterile dH_2_O for a total reaction volume of 25 μL. Amplification was carried out following the program: (i) one cycle of 95°C for 12 min, (ii) 40 cycles of (a) 95°C for 20 s, (b) 60°C for 30 s, and (c) 72°C for 40 s, (iii) dissociation curve. Amplification curves from qPCR were used to decide the optimal number of cycles for each library to reach the PCR amplification plateau, yet without overamplifying the libraries to avoid excessive clonality.

Subsequently, sequencing libraries were PCR-amplified using unique dual-index Illumina primers per sample. PCR reaction was 5 μL 10x PCR Gold Taq buffer, 5 μL MgCl_2_ (25 mM), 0.4 μL dNTP mix (10 mM each), 1 μL (10 μM) for each P7 and P5 primers, 1 μL AmpliTaq GOLD DNA polymerase, 26.6 μL sterile dH_2_O, and 10 μL sample library for a total reaction volume of 50 μL. PCR cycling conditions were as follows: 1 cycle at 95°C for 12 min; 7–20 cycles of 95°C for 20 s, 60°C for 30 s, and 72°C for 40 s; 1 cycle at 72°C for 5 min; hold at 4°C. Each sample was amplified for a different number of cycles (7–20), as determined by the previous qPCR assay.

The indexed libraries were purified with SPRI beads using 1:2 bead-to-library ratio. The mixture was incubated for 5 min at room temperature (ca. 21°C), followed by a double wash with 80% ethanol on a 96S Super Magnet (Alpaca, SKU: A001322). The purified DNA was eluted in EBT solution by incubating at 37°C for 10 min. Purified indexed libraries were pooled together and sequenced on an Illumina NovaSeqX platform, aiming for 5 Gb (~17 million reads) of data per sample.

### Bioinformatic data processing

Raw sequencing data were processed using the standard EHI bioinformatic pipeline [33]. Briefly, reads were quality trimmed using fastp [34] before being mapped using Bowtie2 [35] to the *Calotriton arnoldi* reference genome (GCA_963921515.1), which was the closest reference genome to *C. asper* available at the time of the analysis. Host reads were subsequently filtered from BAM files using samtools [36]. Non-host reads were then assembled both individually and coassembled per stream using MEGAHIT [37]. Single-coverage or multi-coverage binning was then performed using CONCOCT [38], Maxbin2 [39] and MetaBAT2 [40], before being refined using MetaWRAP’s bin_refinement module [41, 42]. Metagenome-assembled genomes (MAGs) were dereplicated at 98% ANI using dRep [43] with MASH [44] and FastANI [45], and non-host reads were mapped to the dereplicated MAG catalogue using Bowtie2, with coverage statistics calculated using CoverM [46].

MAGs were quality-checked with CheckM2 [47], and annotated taxonomically using GTDBtk [48] with the GTDB r214 [49]. The phylogenetic tree of MAGs was generated through pruning the reference GTDB genomes used for phylogenetic placement. DRAM [50] was used to annotate MAG functions using multiple reference databases [51–58]. Subsequently, gene annotations were distilled into Genome-Inferred Functional Traits (GIFTs) using distillR [59], producing biologically meaningful annotations that highlight each bacterial genome’s potential to degrade or synthesize compounds relevant to host metabolism. DistillR utilizes a curated database of over 300 metabolic pathways, employing KEGG and Enzyme Commission (EC) identifiers to calculate standardized GIFT values (Table S1). These values range from 0 to 1, where 0 signifies the absence of all genes associated with a particular metabolic pathway, and 1 indicates the presence of all necessary genes. For example, if a pathway step requires two specific identifiers, it is deemed complete when both are present, half-complete when only one is present, and empty if neither is present. In the case of single-locus traits, the GIFT becomes binary. The domains considered encompass functions relevant for both gut (e.g. polysaccharide degradation, vitamin biosynthesis) and skin (e.g. toxin biosynthesis) microbiomes.

The fraction of bacterial and archaeal DNA in our samples was estimated using SingleM’s prokaryotic_fraction function [60, 61]. SingleM estimates the expected genomic data from prokaryotic origin in a sample by measuring the coverage of prokaryotic single-copy marker genes, and inferring genome sizes based on the closest relatives present in public databases. The Domain-Adjusted Mapping Rate (DAMR) was calculated as the rate of read mapping to the MAG catalogue divided by the fraction of the community predicted to be bacterial or archaeal.

Dietary analysis was conducted using BarcodeMapper [62], which quantifies plant, fungal, and animal DNA from shotgun sequencing datasets by mapping reads to taxonomically annotated COI and ITS marker gene sequences. Pairwise microdiversity dissimilarity metrics were computed following the population-ani method implemented in lorikeet [63].

Genomic differences between animals from Atlantic montane streams and Pyrenean subalpine brooks were analysed using kmer-based genome skimming, due to the impossibility of generating sufficient data for variant calling based on the large size of *Calotriton* genomes. Using a custom-made pipeline (https://github.com/alberdilab/genome_skimming), we extracted the sequencing reads mapping to *C. arnoldi* and analysed them using skmer [64], which enables computing distances between sequence read pools based on their combined kmer profiles. After filtering out samples with fewer than 5 million reads mapped to the reference genome to reduce noise, we tested for significant differences between the contrasted populations using PERMANOVA.

### Metagenome-assembled genomes diversity and statistical analysis

All statistical analyses were conducted using R software v.4.3.2 [65], and were compiled as a Rmarkdown website enabling full reproduction. We calculated the alpha diversities of microbial communities using Hill numbers [66]. To capture the effects of different diversity components (richness, neutral, phylogenetic) and diversity orders (q = 0 considers only presence/absence, while q = 1 gives weight to MAGs based on their relative abundances), we calculated species richness at q = 0, neutral diversity at q = 1, phylogenetic diversity at q = 1 using the package Hilldiv2 [67]. Alpha diversity differences were determined by a parametric t-test when the data were normally distributed and when the variances between contrasted groups were equal. When these assumptions were not held, a non-parametric Wilcoxon test was performed.

We calculated compositional dissimilarities across samples using Hill numbers by computing the Jaccard-type turnover for neutral and phylogenetic beta diversities at order q = 1 using hilldiv::hillpair. To visualize the variation in microbial composition, we performed nonmetric multidimensional scaling (NMDS) ordination plots based on the derived distance matrices. Differences in dispersion within sampling methods were assessed using the betadisper function in the vegan package [68]. To test for differences in microbial composition between samples, we conducted a PERMANOVA using the adonis2 function in vegan and pairwise comparisons using pairwiseAdonis [69]. Microbial differential abundance analysis was performed using ANCOM-BC2 [70]. Additionally, to visualize bacteria according to their functional traits, MAGs were ordinated based on their GIFTs through PCoA. In this ordination, MAGs with similar functional traits are displayed closer to each other while MAGs with distinct profiles are placed further away. To analyse functional differences, community-weighted averages of GIFTs were calculated using distillR::to.community, which weights each MAG’s traits by its abundance in the sample. Differences between environments were calculated using the Kruskal–Wallis test, followed by the Bonferroni–Holm method for multiple comparisons to obtain adjusted *P*-values.

To assess the influence of bacteria with different levels of prevalence, we partitioned MAGs into three fractions. Genomes detected in all four streams were considered core microbes. Genomes detected in both streams within the same environment, with a minimum prevalence of 20%, were classified as endemic bacteria. Genomes detected at only one environment and with <20% prevalence were considered marginal.

## Results

We captured 31 adult male Pyrenean brook salamanders (*C. asper*) from two Pyrenean subalpine brooks (*n* = 8 + 7) and Atlantic montane streams (*n* = 7 + 9). Individuals from Pyrenean subalpine brooks exhibited significantly greater snout–vent lengths (t-test: t = 2.09, *P*-value = .046). However, no significant differences in weight were detected between environments (t-test: t = 0.82, *P*-value = .419). Given the current limitations to reliably characterize and validate the representativeness of other members of the microbiome (e.g. fungi, viruses), we limited our analysis to bacterial and archaeal components.

### Bacterial genome catalogues associated with *C. asper*

**Figure 2:**
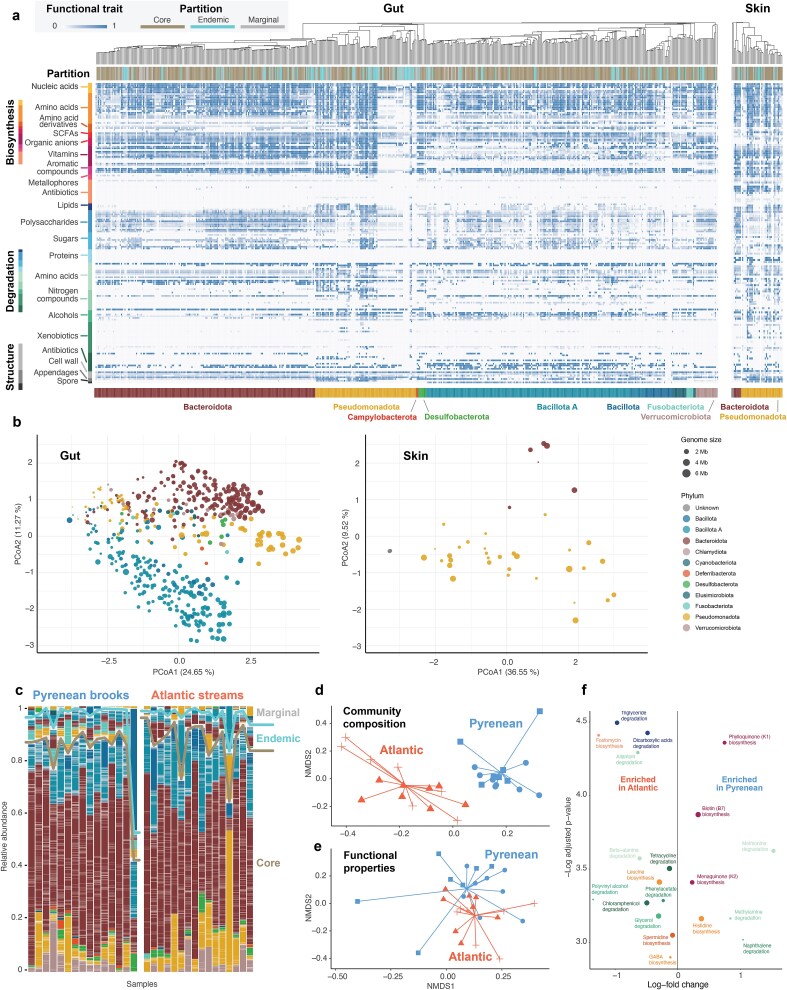
Composition and variation of the gut and skin microbiomes. (a) Phylogeny, partition, and functional characterization of microbial genomes reconstructed from faecal (gut) and skin samples. Each tile represents a metabolic function, with lighter shades indicating lower capacity to accomplish it and darker shades indicating higher capacity. (b) Two-dimensional PCoA ordinations of gut and skin MAGs based on their functional traits. (c) Relative abundances of gut microbiomes in animals from Pyrenean subalpine brooks and Atlantic montane streams, sorted by MAG partitioning into core, endemic and marginal fractions. (d) Compositional dissimilarity of the gut microbiome across animals from Pyrenean subalpine brooks (blue) and Atlantic montane streams (red). Shapes indicate different streams. (e) Functional dissimilarity of the gut microbiome across animals from Pyrenean subalpine brooks (blue) and Atlantic montane streams (red). Shapes indicate different streams. (f) Enrichment of gut microbiome’s metabolic functions in samples from Pyrenean subalpine brooks (left) and Atlantic montane streams (right).

We produced a total of 154.38 and 162.04 Gb (gigabases) of metagenomic data from faecal samples and skin swabs to characterize the gut and skin microbiomes of salamanders. From these data, we generated two reference bacterial genome catalogues (gut and skin) with significantly different features (Fig. 2a). The gut genome catalogue included 539 MAGs, with a mean completeness of 85.01 ± 16.2% and a contamination level of 1.68 ± 2.25% (Fig. S1). An average of 3.66 ± 1.24 Gb out of the 4.98 ± 1.49 Gb sequenced per sample was mapped against the reference catalogue (Fig. S2), yielding a near-complete (99.94 ± 0.24%) estimated recovery of bacterial and archaeal genomes (Fig. S3). In contrast, despite a nearly identical sequencing effort, the skin genome catalogue only recovered 43 genomes. The reconstructed MAGs had a mean completeness of 77.24 ± 17.04% and a contamination level of 4.48 ± 2.42% (Fig. S4). Only 0.25 ± 0.38 Gb out of the 4.47 ± 2.21 Gb of data sequenced per sample mapped against the genome catalogue (Fig. S5), with an estimated microbial fraction recovery of 54.18 ± 12.63% (Fig. S6).

The gut genome catalogue comprised 13 bacterial phyla, with Bacteroidota (56.02 ± 16.86%), Bacillota A (17.72 ± 6.39%), and Pseudomonadota (11.57 ± 13.50%) being the most abundant, collectively accounting for 84.05% of MAGs (Fig. S7). Among the 539 MAGs, 91.65% lacked species-level annotation, and 24.6% lacked genus-level annotation. Species-level annotations were observed in only three phyla: Pseudomonadota (48.28% of MAGs with species-level annotation), Fusobacteriota (33.33%), and Bacillota (4.35%), while the rest of the recovered genomes likely represent previously undescribed taxa. The dereplicated MAG catalogue contained 1 219 667 redundant genes, of which 942 120 (77.24%) were annotated and 557 611 (59.19%) were assigned KEGG orthologs. The functional ordination showed a strong phylogenetic signal: genomes belonging to the same phyla aggregated together, though with significant dispersion within the main clades (Fig. 2b).

The skin genome catalogue comprised bacteria from only two phyla: Pseudomonadota—35 MAGs accounting for 84.9 ± 15.8% of the sequencing reads—and Bacteroidota—six MAGs capturing 14.9 ± 15.8% (Fig. S8). Among the 43 MAGs, 76.74% lacked species-level annotation, and 11.63% lacked genus-level annotation. The MAG catalogue contained 114 303 redundant genes, of which 88 006 (76.99%) were annotated and 52 991 (60.21%) were assigned KEGG orthologs. The functional ordination showed that skin bacteria encompassed a narrower functional landscape compared to gut bacteria, despite the large functional variability within Pseudomonadota (Fig. 2b). Two genomes with very low completeness failed to get phylum-level annotation, but clustered with Pseudomonadota in the functional ordination, suggesting functional similarity despite the lack of taxonomic information.

### Microbiota differences between environments

The gut microbiota of salamanders exhibited comparable alpha diversity values across environments (LM: t-value 1.27, *P*-value = .33, Fig. S9). However, beta diversity analyses revealed distinct community compositions between environments (PERMANOVA-neutral: R^2^ = 0.234, *P*-value = .001; Fig. 2d). A total of 180 MAGs were differentially abundant between environments, yet without consistent enrichment patterns of major microbial lineages. While the average metabolic capacities of the bacterial communities were nearly identical in both environments (Wilcoxon: W = 149, *P*-value = .137), the functional properties of microbiomes differed significantly (PERMANOVA: R^2^ = 0.90, *P*-value = .022; Fig. 2e). Gut microbiomes of Atlantic animals showed significantly higher biosynthetic and degradation capacities of multiple metabolites recognized as relevant for hosts, such as leucine, allantoin, or phenylacetate (Fig. 2f).

To determine whether diet contributed to the observed faecal microbiome differences, we examined the non-bacterial, non-host fraction of our metagenomic data, which contained remnants of invertebrates, vertebrates, plants, and fungi—all potential contributors to gut community composition via direct consumption or secondary ingestion (Fig. S10). However, this snapshot did not reveal any significant variation in host diet between environments (Wilcoxon: W = 101.5, *P*-value = .475; Fig. S11). Similarly, the genome skimming analysis also failed to reveal significant genetic differences between animals from different environments (PERMANOVA: R^2^ = 0.05, F = 0.915, *P*-value = .715).

The skin microbiota of salamanders from Pyrenean subalpine brooks displayed increased richness compared to their Atlantic counterparts (Wilcoxon: *P* = .007), while neutral and phylogenetic alpha diversities remained consistent across environments (Fig. S12). Beta diversity analyses revealed distinct community compositions, with stream-specific effects observed in the Atlantic montane streams (PERMANOVA-neutral: environment, R^2^ = 0.151, *P*-value = .001; stream, R^2^ = 0.111, *P*-value = .018). Accordingly, 10 MAGs were differentially abundant between Pyrenean subalpine brooks and Atlantic montane streams. Functional trait analysis revealed significant differences between the microbiota of salamanders from both environments. The overall metabolic capacity was higher in Atlantic montane streams (0.385 ± 0.0503) compared to animals from Pyrenean subalpine brooks (0.326 ± 0.0525) (Wilcoxon: W = 183, *P* < .001), with multiple metabolic functions showing environment-specific patterns (Fig. S13).

### Contribution of microbiota fractions to functional differences

**Figure 3:**
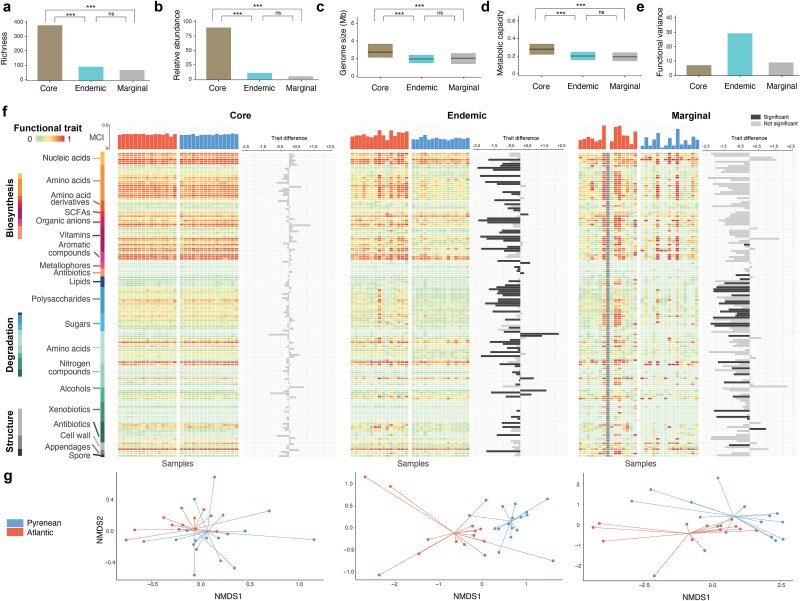
Microbiome partitioning into core, endemic and marginal fractions. (a) Number of genomes assigned to the different fractions. (b) Average cumulative relative abundance of the genomes. (c) Average size of the genomes. (d) Average metabolic capacity of the genomes. (e) Percentage of functional variation explained by the genomes. (f) Community-weighted averages of genome-inferred metabolic traits, partitioned by core, endemic, and marginal fractions. Heatmaps display mean trait values, with darker shades of red indicating greater capacity to carry out the specific function; bar plots above each heatmap show the overall metabolic capacity index (MCI) per sample (the mean across all traits) in Atlantic montane streams (red) and Pyrenean subalpine brooks (blue). Horizontal bar plots indicate the difference between mean values in each environment, with dark colours indicating statistically significant differences between environments. (g) NMDS ordinations of the samples ordinated according to the community-weighed functional properties of core, endemic, and marginal microbiota fractions.

To explore the sources of functional capacity differences, we partitioned the microbial communities into core, endemic, and marginal fractions, according to the prevalence of each genome across streams and elevations. For our analyses, the “core” microbiota referred to MAGs present in samples from all four streams. This core encompassed 389 MAGs (72.2%) (Fig. 3a). The endemic fraction—MAGs present at only one environment—contained 99 genomes (18.3%). Finally, the marginal fraction contained bacteria sparsely represented in only a few individuals; it consisted of 51 MAGs (9.5%). In terms of relative abundances, the averaged aggregated representation of core bacteria across all animals was 87%, followed by 8.8% of endemic and 4.1% of marginal bacteria (Fig. 3b). The average genome size (Wilcoxon: W = 21 740, p.adj < 0.001; Fig. 3c) and metabolic capacity (Wilcoxon: W = 21 482, p.adj < 0.001; Fig. 3d) of core bacteria were significantly higher than those belonging to the endemic and marginal fractions. Functional capacity differences between core and endemic bacteria were mainly driven by greater genomic potential for organic anion and vitamin biosynthesis, as well as degradation of polysaccharides and amino acids. In addition, the functional attributes of Pyrenean and Atlantic endemic bacteria also differed significantly, with bacteria endemic to Atlantic montane streams displaying higher overall metabolic capacities (Wilcoxon: W = 552, *P* < .002), with increased capacities for a number of functions, including amino acid biosynthesis, sugar degradation, and antibiotic degradation.

These differences between core and endemic bacteria, along with variations in endemisms, influenced how bacteria contribute to the functional differences between communities in animals from different environments (Fig. 3f). Core and marginal bacteria both exhibited differences between environments (*P*-value = .003 and *P*-value = .014, respectively), though the variance explained was modest (11.9% and 11.1%). No GIFTs differed significantly between environments in the core microbiota, whereas 34 GIFTs did so in the marginal fraction (Fig. 3e). The endemic fraction demonstrated the largest effect size, with an R^2^ of 0.29 and 89 GIFTs with differing values between Pyrenean subalpine brooks and Atlantic montane streams, resulting in a highly significant difference between environments (*P*-value <.001) (Fig. 3g).

In the case of the skin, the core microbiome encompassed 25 MAGs (58%), while the endemic fraction contained 7 (16%) and the marginal fraction 11 (25.6%). Considered together, on average, the core bacteria represented 90% of aggregate relative abundance, followed by 13.2% of endemic and 8.7% of marginal bacteria. The low number of genomes per category precluded us from conducting more detailed analyses.

### Microdiversity differences between environments

**Figure 4:**
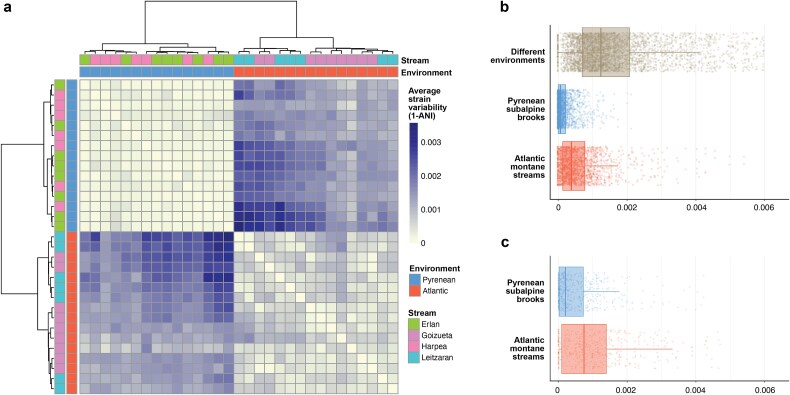
Genome microdiversity. (a) Heatmap of average pairwise microdiversity (1—ANI) among core genomes across all samples, sorted by hierarchical clustering. Darker tiles indicate larger variability between microbial genomes across samples. (b) Average pairwise microdiversity of core genomes between samples from different environments, among samples from Pyrenean subalpine brooks, and among samples from Atlantic montane streams. (c) Average pairwise microdiversity of endemic genomes, comparing Pyrenean versus Atlantic samples.

To assess strain-level variability among gut bacteria, we compared reconstructed genomes across environments and microbiome fractions. Although both environments harboured the same core bacterial species, strains isolated within the same environment exhibited higher average nucleotide identity (ANI) to one another than to strains from the other environment, highlighting microdiversity differences between environments (Fig. 4a). Moreover, strains from Atlantic montane streams showed significantly greater microdiversity than their Pyrenean counterparts (Wilcoxon: W = 8720, *P*-value <.001; Fig. 4b). A similar pattern was observed among endemic taxa: microdiversity values of Atlantic endemics exceeded those of Pyrenean endemics (W = 70 662, *P*-value <.001; Fig. 4c). Together, these results indicate that—despite comparable overall gut-microbiome diversity across environments—within-species microdiversity was greater at Atlantic montane streams.

## Discussion

This first comprehensive characterization of the gut and skin microbiota of the Pyrenean brook salamander (*C. asper*) unveiled unique insights into the structure and source of functional microbiome variation across populations and environments.

### Uncovering a concealed diversity

To date, the majority of studies profiling amphibian gut and skin microbiotas have relied on 16S amplicon sequencing [71–75]. While this approach has provided an initial glimpse into microbiota composition and structure [76], it is limited by existing sequence diversity, as it relies on previously sequenced and taxonomically annotated 16S rRNA gene references [77]. In contrast, our results highlight how genome-resolved shotgun metagenomics enables the discovery of previously uncharacterized taxa and their corresponding functional capacities, providing a more comprehensive view of host–microbiota relationships and advancing our understanding of microbial ecology in wildlife. Using this approach, we successfully reconstructed 582 bacterial genomes spanning 13 phyla, over 90% of which exhibited an ANI below 95% relative to any genome indexed in current databases. These results suggest that a large proportion of bacterial species associated with *C. asper* are not represented in current reference databases [78, 79], underscoring how much remains to be discovered about the bacterial diversity of newts and salamanders.

The vast majority (539) of reconstructed bacterial genomes derived from faecal samples, with only 43 genomes reconstructed from skin swab samples. While our DAMR analyses indicated near-complete reconstruction of the bacterial genomes present in faecal samples, the same could not be achieved for skin samples. We estimated that nearly half of the bacterial genomic information in the skin samples was likely not represented in our MAG catalogue. This limitation arises because skin swabs typically contain large proportions of host DNA, often exceeding 90% of all reads, together with a diverse array of low-abundance environmental bacteria. Both factors substantially increase sequencing requirements and complicate metagenomic assemblies [80]. The challenge of recovering skin microbiomes is further compounded by the significantly lower amount of DNA present in skin samples compared to faecal samples, which increases the complexity of sequencing library preparation and determines the quality of sequencing [81]. In any case, the pronounced disparity in DAMR values between faecal and skin samples highlights the critical importance of assessing genome recovery success in genome-resolved metagenomics, ensuring the accurate interpretation of results [60].

Despite these caveats, the recovered bacterial communities were markedly distinct. While we cannot entirely rule out the reconstruction of some environmental bacteria, the bacterial communities derived from faecal samples clearly resembled a typical vertebrate gut microbiome, characterized by a high diversity of anaerobic bacteria [82]. This pattern indicates that our careful sampling procedures were effective in capturing the gut-associated microbial community while minimizing the contribution of environmental bacteria. The gut microbiome of *C. asper* was dominated by Bacteroidota, Bacillota A, and Pseudomonadota. These phyla were also identified as the most broadly represented in the North American eastern newt (*Notophthalmus viridescens)* [83], suggesting that they may represent a coarse-scale gut microbiota typical of urodeles. The skin microbiome differed markedly from the gut microbiome, displaying significantly lower diversity and an overwhelming dominance of Pseudomonadota. Despite the aforementioned limitations in reconstructing bacterial genomes from skin swabs, the recovered community—dominated by Gammaproteobacteria, Bacteroidia, and Alphaproteobacteria—closely resembled those reported in other newts and salamanders [84, 85].

### Different microbiomes at different environments

The distinct microbial community structures observed between animals from Atlantic montane streams and Pyrenean subalpine brooks suggest that habitat differences play a significant role in shaping the gut microbiota of *C. asper*. Gut microbiome differences neither affected overall alpha diversity nor metabolic capacity values between habitat types. However, community rearrangements resulted in significant changes in the metabolic properties of the bacterial communities, with significant enrichment in various functions. Notably, these included biosynthesis of several amino acids (e.g. arginine, lysine, phenylalanine, and tryptophan) and their derivatives, as well as enhanced degradation of lipids, polysaccharides, and nitrogen compounds. We hypothesized that these changes could be indicative of a broader dietary niche in salamanders inhabiting Atlantic montane streams, with an expanded exposure to nutrients that produce more metabolic niches for bacteria [86, 87]. However, our dietary analyses did not reveal any notable difference in the coarse-scale dietary items, which were dominated by arthropods and annelids at both environments, with a significant proportion of Tremellales fungi and traces of fabaceus trees, plausibly derivative from ingesting detritus. Regardless of active predation or passive ingestion, these taxa contain different nutrients that could shape gut microbiomes, but their distribution did not show a consistent bias towards a single stream or environment. Interestingly, skin microbiomes, which are not directly related to nutrition, also displayed the same pattern, suggesting that factors other than diet drive the functional differences between environments.

Considering that microbiome profiles often mirror host genomic differences [88, 89], and given that our Atlantic population has not been included in the genetic analysis of *C. asper* [28], we tested whether kmer-based genome skimming of host reads recovered from skin swab samples revealed significant differences between animals from Atlantic montane streams and Pyrenean subalpine brooks. While the resolution of the employed approach is limited, the absence of a significant signal suggests that the genetic differences between the contrasted populations are not drastic, reducing the likelihood that the observed changes were due to genetic differences.

### The contribution of endemic bacteria to functional variation

To further investigate these functional differences, we traced their origins in the gut microbiomes of Pyrenean and Atlantic *C. asper* by categorizing the bacteria into core, endemic, and marginal groups. The majority (389 MAGs) were classified as part of the core microbiome. This suggests that, despite geographical separation, genetic isolation of the host populations, and environmental variation, a substantial portion of the *C. asper* microbiome remains consistently associated with the host. These core bacteria constituted the bulk of the microbiome, typically accounting for 80%–90% of sequencing reads, indicating a notably consistent core microbiome, which in other systems has often been associated with host stability or health [90]. A significant subset of bacteria (99 MAGs) was classified as endemic, being both exclusive to and widespread within individuals from only a single environment, and representing 10%–15% of the microbiome. Almost the entire diversity of Fusobacteriota—characterized by a high capacity for amino acid degradation—was restricted to endemic bacteria from Atlantic montane streams, contributing to the functional distinctiveness of microbiomes. Lastly, marginal bacteria (51 MAGs), found in only a few individuals within one population, emphasized the stochastic nature of microbiomes in wild animals. Typically comprising <5% of the microbiome, these bacteria reflect individual-specific variation not representative of the broader population, though additional sampling is required to confirm their low prevalence across the host population.

Partitioning the microbiota also revealed significant differences in the functional properties of the genomes associated with each fraction. We hypothesized that the core bacterial fraction would exhibit smaller average genome sizes, based on the assumption that it comprises taxa closely associated with the host and subject to substantial (pseudo)vertical transmission. Such transmission would be expected to favour bacteria with reduced genomes and limited metabolic capabilities, relying on interactions with other taxa for survival [91, 92]. Contrary to our expectations, the average genome size of core bacteria was not smaller, but in fact significantly larger than that of endemic and marginal taxa. These findings point to an alternative scenario: bacteria with reduced genome size and high metabolic dependency may experience less extensive (pseudo)vertical transmission, as their successful colonization likely depends on the prior establishment of a structured microbial community capable of providing metabolic by-products essential for their survival [93].

These results also challenge our prior assumption that marginal bacteria are merely opportunistic colonizers, entering gut microbial communities through sporadic events. The similarity in functional profiles between marginal and endemic bacteria suggests that both fractions are largely composed of taxa that integrate into an already complex microbiome, primarily structured by core taxa. These core taxa appear to generate certain metabolic niches more frequently than others, with endemic bacteria occupying the more persistent niches and marginal bacteria associated with the rarer ones [9]. As a result, the ecological significance of marginal bacteria may be greater than previously assumed.

### Increased metabolic capacity and microdiversity at montane streams compared to subalpine brooks

The functional and microdiversity analyses comparing animals across both environments revealed similar patterns. The average metabolic capacity of endemic and marginal taxa from Atlantic animals was higher than that of their Pyrenean counterparts, reflecting the broader observation that skin microbes from Atlantic animals exhibited significantly greater overall metabolic capacities. Additionally, gut bacteria from Atlantic montane stream salamanders displayed higher intraspecific variability than those at Pyrenean subalpine brooks.

While bacterial community diversity analyses are widespread, to our knowledge, no studies have yet characterized community-level variation in genome sizes, metabolic capacities, and microdiversity across environments. Traditional diversity studies in freshwater systems have produced highly context-dependent results, with diversity found to correlate both positively and negatively with elevation [94], largely influenced by regional habitat complexity [95]. Although logistical constraints prevented us from sampling water, detritus, and dietary animal sources—limiting direct comparisons between environmental and gut microbiomes—the consistent patterns observed in both skin and gut bacteria suggest the existence of an environmental microbial pool at Atlantic montane streams with enhanced metabolic capacity and greater strain-level variability compared to Pyrenean subalpine brooks.

These patterns may reflect the higher energy availability, environmental complexity, and overall biodiversity typical of low-elevation streams, which could promote bacterial diversification and functional enrichment [96]. This complexity, in turn, may be mirrored in the microbiomes of salamanders inhabiting these environments. In our study area, Atlantic montane streams located at lower elevation were characterized by a more structurally complex landscape, comprising a heterogeneous mosaic of forests and small agricultural plots. In contrast, Pyrenean subalpine brooks were situated in more uniform open meadows.

### Potential impact of functional differences on salamander biology

Ultimately, variation in the presumed availability and recruitment of bacteria with diverse functional capacities across environments may influence salamander biology—potentially enhancing nutrient extraction and energy acquisition from different dietary sources. Gut microbiomes of salamanders from Pyrenean subalpine brooks were enriched for B7, K1, and K2 vitamin biosynthesis pathways, as previously observed in reptiles and mammals at high elevations [97, 98]. The higher vitamin biosynthesis capacity may help offset dietary vitamin deficiencies in a nutritionally poorer environment and potentially contribute to the larger body sizes observed at high-elevation sites [99, 100]. Although animals from Pyrenean subalpine brooks exhibited significantly greater snout–vent lengths, no significant differences in weight were observed, suggesting increased fat accumulation in Atlantic individuals inhabiting lower elevations. This could be supported by the two microbial functions most enriched in Atlantic animals—triglyceride and dicarboxylic acid degradation—both associated with lipid metabolism. While the observed functional differences in the microbiome correspond with phenotypic variation, experimental manipulation will be necessary to determine whether microbial properties causally influence salamander physiology.

## Conclusion

Our study provides the first comprehensive catalogue of bacterial genomes associated with wild salamanders and reveals strong ecological patterns through analyses of microbiota partitioning, metabolic capacity, and microdiversity patterns that were not captured by traditional diversity metrics or taxonomic enrichment analyses. Our results demonstrate that the functional differences across environments were largely driven by endemic bacteria recruited locally. In addition, both functional capacities and intraspecific microdiversity of bacteria differed between contrasting environments, with salamanders from low-elevation Atlantic streams harbouring more diverse and functionally richer microbiomes than their Pyrenean counterparts. The consistency of these patterns in both gut and skin microbiomes suggests that environment-specific factors shape the pool of available bacterial functions, raising the question of whether and how salamanders selectively filter this pool to assemble gut and skin microbiomes that are at least neutral—or potentially beneficial—to their fitness.

Our work, conducted as part of the EHI [27], demonstrates the feasibility of applying genome-resolved metagenomic analyses to wild vertebrates, including the herpetofauna [80]. Amphibians and reptiles have proven well-suited for such investigations, which can yield deep insights into the functional roles of host-associated microorganisms. Ultimately, this knowledge may prove instrumental in understanding the conservation status of amphibian species and improving captive breeding and translocation efforts aimed at reversing global declines.

## Supplementary Material

ycag106_supplementary_material_final

## Data accessibility

The raw data and microbial genome sequences were published as part of the 1st EHI Data Release [101], under BioProject PRJEB76898. The full code that enables reproducing all the analyses included in this manuscript, as well as the accession numbers of the raw data sets, can be found in a dedicated GitHub repository (https://github.com/alberdilab/calotriton_metagenomics), with a snapshot stored in Zenodo with doi: 10.5281/zenodo.16908317.

## References

[1] Ruuskanen MO, Sommeria-Klein G, Havulinna AS et al. Modelling spatial patterns in host-associated microbial communities. *Environ Microbiol* 2021;23:2374–88. 10.1111/1462-2920.15462 33734553

[2] Yatsunenko T, Rey FE, Manary MJ et al. Human gut microbiome viewed across age and geography. *Nature* 2012;486:222–7. 10.1038/nature11053 22699611 PMC3376388

[3] Ang L, Vinderola G, Endo A et al. Gut microbiome characteristics in feral and domesticated horses from different geographic locations. *Commun Biol* 2022;5:172. 10.1038/s42003-022-03116-2 35217713 PMC8881449

[4] Kwong WK, Medina LA, Koch H et al. Dynamic microbiome evolution in social bees. *Sci Adv* 2017;3:e1600513. 10.1126/sciadv.1600513 28435856 PMC5371421

[5] Mancabelli L, Milani C, Lugli GA et al. Meta-analysis of the human gut microbiome from urbanized and pre-agricultural populations. *Environ Microbiol* 2017;19:1379–90. 10.1111/1462-2920.13692 28198087

[6] Hooper R, Brealey JC, van der Valk T et al. Host-derived population genomics data provides insights into bacterial and diatom composition of the killer whale skin. *Mol Ecol* 2019;28:484–502. 10.1111/mec.14860 30187987 PMC6487819

[7] Amato KR, Yeoman CJ, Kent A et al. Habitat degradation impacts black howler monkey (Alouatta pigra) gastrointestinal microbiomes. *ISME J* 2013;7:1344–53. 10.1038/ismej.2013.16 23486247 PMC3695285

[8] Louca S, Polz MF, Mazel F et al. Function and functional redundancy in microbial systems. *Nat Ecol Evol* 2018;2:936–43. 10.1038/s41559-018-0519-1 29662222

[9] Malard LA, Guisan A. Into the microbial niche. *Trends Ecol Evol* 2023;38:936–45. 10.1016/j.tree.2023.04.015 37236880

[10] Moya A, Ferrer M. Functional redundancy-induced stability of gut microbiota subjected to disturbance. *Trends Microbiol* 2016;24:402–13. 10.1016/j.tim.2016.02.002 26996765

[11] Koziol A, Odriozola I, Leonard A et al. Mammals show distinct functional gut microbiome dynamics to identical series of environmental stressors. *MBio* 2023;14:e0160623. 10.1128/mbio.01606-23 37650630 PMC10653949

[12] Fontaine SS, Mineo PM, Kohl K. Experimental manipulation of microbiota reduces host thermal tolerance and fitness under heat stress in a vertebrate ectotherm. *Nat Ecol Evol* 2022;6:405–17. 10.1038/s41559-022-01686-2 35256809

[13] Bletz MC, Perl RGB, Bobowski BT et al. Amphibian skin microbiota exhibits temporal variation in community structure but stability of predicted bd-inhibitory function. *ISME J* 2017;11:1521–34. 10.1038/ismej.2017.41 28387770 PMC5520157

[14] Kueneman JG, Parfrey LW, Woodhams DC et al. The amphibian skin-associated microbiome across species, space and life history stages. *Mol Ecol* 2014;23:1238–50. 10.1111/mec.12510 24171949

[15] Wagener C, du Plessis M, Measey J. Invasive amphibian gut Microbiota and functions shift differentially in an expanding population but remain conserved across established populations. *Microb Ecol* 2022;84:1042–54. 10.1007/s00248-021-01896-4 34735604

[16] Bates KA, Sommer U, Hopkins KP et al. Microbiome function predicts amphibian chytridiomycosis disease dynamics. *Microbiome* 2022;10:44. 10.1186/s40168-021-01215-6 35272699 PMC8908643

[17] Kang X, Shao M, Jiang J et al. The gut microbiome of the Asiatic toad (Bufo gargarizans) reflects environmental changes and human activities. *Ecol Evol* 2025;15:e71394. 10.1002/ece3.71394 40342698 PMC12058643

[18] Martínez-Ugalde E, Ávila-Akerberg V, González Martínez TM et al. Gene functions of the Ambystoma altamirani skin microbiome vary across space and time but potential antifungal genes are widespread and prevalent. *Microb Genom* 2024;10:001181. 10.1099/mgen.0.001181 38240649 PMC10868611

[19] Gupta VK, Paul S, Dutta C. Geography, ethnicity or subsistence-specific variations in human microbiome composition and diversity. *Front Microbiol* 2017;8:1162. 10.3389/fmicb.2017.01162 28690602 PMC5481955

[20] Neu AT, Allen EE, Roy K. Defining and quantifying the core microbiome: challenges and prospects. *Proc Natl Acad Sci USA* 2021;118:e2104429118. 10.1073/pnas.2104429118 34862327 PMC8713806

[21] Risely A . Applying the core microbiome to understand host-microbe systems. *J Anim Ecol* 2020;89:1549–58. 10.1111/1365-2656.13229 32248522

[22] Asnicar F, Manara S, Zolfo M et al. Studying vertical microbiome transmission from mothers to infants by strain-level metagenomic profiling. *mSystems* 2017;2:e00164-16. http://paperpile.com/b/F3Xd9S/eV5kK. 10.1128/mSystems.00164-16 PMC5264247 28144631

[23] Nyholm SV, McFall-Ngai M. A lasting symbiosis: how the Hawaiian bobtail squid finds and keeps its bioluminescent bacterial partner. *Nat Rev Microbiol* 2021;19:666–79. 10.1038/s41579-021-00567-y 34089010 PMC8440403

[24] Michel A, Minocher R, Niehoff P-P et al. Isolated Grauer’s gorilla populations differ in diet and gut microbiome. *Mol Ecol* 2023;32:6523–42. 10.1111/mec.16663 35976262

[25] Alberdi A, Limborg MT, Groussin M et al. Metagenomic spaces: a framework to study the effect of microbiome variation on animal ecology and evolution. *J Evol Biol* 2025;38:1285–98. 10.1093/jeb/voaf063 40402820

[26] Kohl KD, Weiss RB, Cox J et al. Gut microbes of mammalian herbivores facilitate intake of plant toxins. *Ecol Lett* 2014;17:1238–46. 10.1111/ele.12329 25040855

[27] Leonard A, Earth Hologenome Initiative Consortium, Alberdi A. A global initiative for ecological and evolutionary hologenomics. *Trends Ecol Evol* 2024;39:616–20. 10.1016/j.tree.2024.03.005 38777633

[28] Valbuena-Ureña E, Oromi N, Soler-Membrives A et al. Jailed in the mountains: genetic diversity and structure of an endemic newt species across the Pyrenees. *PLoS One* 2018;13:e0200214. 10.1371/journal.pone.0200214 30071027 PMC6071966

[29] Hyatt AD, Boyle DG, Olsen V et al. Diagnostic assays and sampling protocols for the detection of Batrachochytrium dendrobatidis. *Dis Aquat Org* 2007;73:175–92. 10.3354/dao073175 17330737

[30] Boyle DG, Boyle DB, Olsen V et al. Rapid quantitative detection of chytridiomycosis (Batrachochytrium dendrobatidis) in amphibian samples using real-time Taqman PCR assay. *Dis Aquat Org* 2004;60:141–8. 10.3354/dao060141 15460858

[31] Lauritsen JG, Carøe C, Gaun N et al. Robust, open-source, and automation-friendly DNA extraction protocol for hologenomic research. *Mol Ecol Resour* 2025;25:e70042. 10.1111/1755-0998.70042 40923293 PMC12550468

[32] Carøe C, Gopalakrishnan S, Vinner L et al. Single-tube library preparation for degraded DNA. *Methods Ecol Evol* 2017;9:410–9. 10.1111/2041-210X.12871

[33] Eisenhofer R, Alberdi A. The earth Hologenome initiative bioinformatics workflow. *The Earth Hologenome Initiative Bioinformatics Workflow* https://www.earthhologenome.org/bioinformatics. Accessed 12 Jan 2023.

[34] Chen S, Zhou Y, Chen Y et al. Fastp: an ultra-fast all-in-one FASTQ preprocessor. *Bioinformatics* 2018;34:i884–90. 10.1093/bioinformatics/bty560 30423086 PMC6129281

[35] Langmead B, Salzberg SL. Fast gapped-read alignment with bowtie 2. *Nat Methods* 2012;9:357–9. 10.1038/nmeth.1923 22388286 PMC3322381

[36] Li H, Handsaker B, Wysoker A et al. The sequence alignment/map format and SAMtools. *Bioinformatics* 2009;25:2078–9. 10.1093/bioinformatics/btp352 19505943 PMC2723002

[37] Li D, Liu C-M, Luo R et al. MEGAHIT: an ultra-fast single-node solution for large and complex metagenomics assembly via succinct de Bruijn graph. *Bioinformatics* 2015;31:1674–6. 10.1093/bioinformatics/btv033 25609793

[38] Alneberg J, Bjarnason BS, de Bruijn I et al. Binning metagenomic contigs by coverage and composition. *Nat Methods* 2014;11:1144–6. 10.1038/nmeth.3103 25218180

[39] Wu Y-W, Simmons BA, Singer SW. MaxBin 2.0: an automated binning algorithm to recover genomes from multiple metagenomic datasets. *Bioinformatics* 2015;32:605–7. 26515820 10.1093/bioinformatics/btv638

[40] Kang DD, Li F, Kirton E et al. MetaBAT 2: an adaptive binning algorithm for robust and efficient genome reconstruction from metagenome assemblies. *PeerJ* 2019;7:e7359. 10.7717/peerj.7359 31388474 PMC6662567

[41] Uritskiy GV, DiRuggiero J, Taylor J. MetaWRAP—a flexible pipeline for genome-resolved metagenomic data analysis. *Microbiome* 2018;6:1–13. 10.1186/s40168-018-0541-1 30219103 PMC6138922

[42] Parks DH, Imelfort M, Skennerton CT et al. CheckM: assessing the quality of microbial genomes recovered from isolates, single cells, and metagenomes. *Genome Res* 2015;25:1043–55. 10.1101/gr.186072.114 25977477 PMC4484387

[43] Olm MR, Brown CT, Brooks B et al. dRep: a tool for fast and accurate genomic comparisons that enables improved genome recovery from metagenomes through de-replication. *ISME J* 2017;11:2864–8. 10.1038/ismej.2017.126 28742071 PMC5702732

[44] Ondov BD, Treangen TJ, Melsted P et al. Mash: fast genome and metagenome distance estimation using MinHash. *Genome Biol* 2016;17:132. 10.1186/s13059-016-0997-x 27323842 PMC4915045

[45] Jain C, Rodriguez-R LM, Phillippy AM et al. High throughput ANI analysis of 90K prokaryotic genomes reveals clear species boundaries. *Nat Commun* 2018;9:5114. 10.1038/s41467-018-07641-9 30504855 PMC6269478

[46] STN Aroney, RJP Newell, J Nissen et al. CoverM: Read Coverage Calculator for Metagenomics. Bioinform 2024; 41:btaf147. 10.1093/bioinformatics/btaf147.

[47] Chklovski A, Parks DH, Woodcroft BJ et al. CheckM2: a rapid, scalable and accurate tool for assessing microbial genome quality using machine learning. *Nat Methods* 2023; 20:1203–12. 37500759 10.1038/s41592-023-01940-w

[48] Chaumeil P-A, Mussig AJ, Hugenholtz P et al. GTDB-Tk: a toolkit to classify genomes with the genome taxonomy database. *Bioinformatics* 2019;36:1925–7. 10.1093/bioinformatics/btz848 31730192 PMC7703759

[49] Rinke C, Chuvochina M, Mussig AJ et al. A standardized archaeal taxonomy for the genome taxonomy database. *Nat Microbiol* 2021;6:946–59. 10.1038/s41564-021-00918-8 34155373

[50] Shaffer M, Borton MA, McGivern BB et al. DRAM for distilling microbial metabolism to automate the curation of microbiome function. *Nucleic Acids Res* 2020;48:8883–900. 10.1093/nar/gkaa621 32766782 PMC7498326

[51] Steinegger M, Söding J. MMseqs2 enables sensitive protein sequence searching for the analysis of massive data sets. *Nat Biotechnol* 2017;35:1026–8. 10.1038/nbt.3988 29035372

[52] Finn RD, Clements J, Eddy SR. HMMER web server: interactive sequence similarity searching. *Nucleic Acids Res* 2011;39:W29–37. 10.1093/nar/gkr367 21593126 PMC3125773

[53] Kanehisa M, Goto S. KEGG: Kyoto encyclopedia of genes and genomes. *Nucleic Acids Res* 2000;28:27–30. 10.1093/nar/28.1.27 10592173 PMC102409

[54] Bateman A, Coin L, Durbin R et al. The Pfam protein families database. *Nucleic Acids Res* 2004;32:138D–41. 10.1093/nar/gkh121 PMC308855 14681378

[55] Huerta-Cepas J, Szklarczyk D, Heller D et al. eggNOG 5.0: a hierarchical, functionally and phylogenetically annotated orthology resource based on 5090 organisms and 2502 viruses. *Nucleic Acids Res* 2019;47:D309–14. 10.1093/nar/gky1085 30418610 PMC6324079

[56] Chan PP, Lowe TM. tRNAscan-SE: searching for tRNA genes in genomic sequences. *Methods Mol Biol* 2019;1962:1–14, New York, NY, Springer New York. 10.1007/978-1-4939-9173-0_1 31020551 PMC6768409

[57] Seemann T, Booth T. Barrnap: basic rapid ribosomal RNA predictor. *GitHub repository* 2018. https://github.com/tseemann/barrnap

[58] Park BH, Karpinets TV, Syed MH et al. CAZymes analysis toolkit (CAT): web service for searching and analyzing carbohydrate-active enzymes in a newly sequenced organism using CAZy database. *Glycobiology* 2010;20:1574–84. 10.1093/glycob/cwq106 20696711

[59] Alberdi A, Langa J. distillR: Distillation of Gene Annotations into Genome-Inferred Functional Traits, 2024. https://github.com/alberdilab/distillR

[60] Eisenhofer R, Alberdi A, Woodcroft B et al. Large-scale estimation of bacterial and archaeal DNA prevalence in metagenomes reveals biome-specific patterns. *mSystems* 2026;**11**;01062–25. 10.1128/msystems.01062-25 PMC13098197 41854267

[61] Woodcroft BJ, Aroney STN, Zhao R et al. Comprehensive taxonomic identification of microbial species in metagenomic data using SingleM and sandpiper. *Nat Biotechnol* 2025;1–6. 10.1038/s41587-025-02738-1 40670710

[62] Alberdi A . BarcodeMapper: https://github.com/alberdilab/barcodemapper, 2025.

[63] Newell R . Bioinformatic Methods for Genome-Centric Metagenomics, 2023.

[64] Sarmashghi S, Bohmann K, Gilbert P et al. Skmer: assembly-free and alignment-free sample identification using genome skims. *Genome Biol* 2019;20:34. 10.1186/s13059-019-1632-4 30760303 PMC6374904

[65] R Core Team R, Others . R: A Language and Environment for Statistical Computing, 2013. The R Core Team

[66] Hill MO . Diversity and evenness: a unifying notation and its consequences. *Ecology* 1973;54:427–32. 10.2307/1934352

[67] Alberdi A, Gilbert MTP. A guide to the application of Hill numbers to DNA based diversity analyses. *Mol Ecol Resour* 2019;19:804–17. 10.1111/1755-0998.13014 30947383

[68] Oksanen J, Blanchet FG, Kindt R et al. Package ‘Vegan’. Community Ecology Package, version, Vol. 2, 2013, https://cran.r-project.org/web/packages/vegan/index.html.

[69] Martinez AP . pairwiseAdonis: Pairwise Multilevel Comparison Using Adonis. R package version 0.4. Github, 2020. https://github.com/pmartinezarbizu/pairwiseadonis

[70] Lin H, Peddada SD. Multigroup analysis of compositions of microbiomes with covariate adjustments and repeated measures. *Nat Methods* 2024;21:83–91. 10.1038/s41592-023-02092-7 38158428 PMC10776411

[71] Barnes EM, Kutos S, Naghshineh N et al. Assembly of the amphibian microbiome is influenced by the effects of land-use change on environmental reservoirs. *Environ Microbiol* 2021;23:4595–611. 10.1111/1462-2920.15653 34190389

[72] Santos B, Martins FMS, Sabino-Pinto J et al. Skin and gut microbiomes of tadpoles vary differently with host and water environment: a short-term experiment using 16S metabarcoding. *Sci Rep* 2023;13:16321. 10.1038/s41598-023-43340-2 37770544 PMC10539280

[73] Muletz Wolz CR, Yarwood SA, Campbell Grant EH et al. Effects of host species and environment on the skin microbiome of plethodontid salamanders. *J Anim Ecol* 2018;87:341–53. 10.1111/1365-2656.12726 28682480

[74] Afonso M, Coelho L, Jesus F et al. Effects of pine and eucalypt ashes on bacterial isolates from the skin microbiome of the fire salamander (Salamandra salamandra). *Sci Total Environ* 2022;841:156677. 10.1016/j.scitotenv.2022.156677 35710008

[75] Bletz MC, Goedbloed DJ, Sanchez E et al. Amphibian gut microbiota shifts differentially in community structure but converges on habitat-specific predicted functions. *Nat Commun* 2016;7:13699. 10.1038/ncomms13699 27976718 PMC5171763

[76] Hernández-Gómez O, Hua J. From the organismal to biosphere levels: environmental impacts on the amphibian microbiota. *FEMS Microbiol Rev* 2023;47:fuad002 http://paperpile.com/b/F3Xd9S/OSY7I. 10.1093/femsre/fuad002 36725211

[77] Yoon S-H, Ha S-M, Kwon S et al. Introducing EzBioCloud: a taxonomically united database of 16S rRNA gene sequences and whole-genome assemblies. *Int J Syst Evol Microbiol* 2017;67:1613–7. 10.1099/ijsem.0.001755 28005526 PMC5563544

[78] Rodriguez-R LM, Conrad RE, Viver T et al. An ANI gap within bacterial species that advances the definitions of intra-species units. *MBio* 2024;15:e0269623. 10.1128/mbio.02696-23 38085031 PMC10790751

[79] Conrad R, Brink CE, Viver T et al. Microbial species and intraspecies units exist and are maintained by ecological cohesiveness coupled to high homologous recombination. *Nat Commun* 2024;15:9906. 10.1038/s41467-024-53787-0 39548060 PMC11568254

[80] Pietroni C, Gaun N, Leonard A et al. Hologenomic data generation and analysis in wild vertebrates. *Methods Ecol Evol* 2024;16:97–107. 10.1111/2041-210X.14456

[81] Aizpurua O, Dunn RR, Hansen LH et al. Field and laboratory guidelines for reliable bioinformatic and statistical analysis of bacterial shotgun metagenomic data. *Crit Rev Biotechnol* 2023;44:1164–82. 10.1080/07388551.2023.2254933 37731336

[82] Levin D, Raab N, Pinto Y et al. Diversity and functional landscapes in the microbiota of animals in the wild. *Science* 2021;372:eabb5352. 10.1126/science.abb5352 33766942

[83] Fontaine SS, Mineo PM, Kohl KD. Changes in the gut microbial community of the eastern newt (Notophthalmus viridescens) across its three distinct life stages. *FEMS Microbiol Ecol* 2021;97:fiab021. 10.1093/femsec/fiab021 33547890

[84] Walke JB, Becker MH, Loftus SC et al. Amphibian skin may select for rare environmental microbes. *ISME J* 2014;8:2207–17. 10.1038/ismej.2014.77 24858782 PMC4992085

[85] Wan B, Chen G, Poon ESK et al. Environmental factors and host sex influence the skin microbiota structure of Hong Kong newt (Paramesotriton hongkongensis) in a coldspot of chytridiomycosis in subtropical East Asia. *Integr Zool* 2024;20:236–255. 10.1111/1749-4877.12855 PMC11897979 38872359

[86] Beck WS, Rugenski AT, Poff NL. Limiting nutrients drive mountain stream ecosystem processes along an elevation gradient. *Freshw Sci* 2021;40:368–81. 10.1086/714441

[87] Valett HM, Crenshaw CL, Wagner PF. Stream nutrient uptake, forest succession, and biogeochemical theory. *Ecology* 2002;83:2888–901. 10.1890/0012-9658(2002)083[2888:SNUFSA]2.0.CO;2

[88] Kurilshikov A, Wijmenga C, Fu J et al. Host genetics and gut microbiome: challenges and perspectives. *Trends Immunol* 2017;38:633–47. 10.1016/j.it.2017.06.003 28669638

[89] Bonder MJ, Kurilshikov A, Tigchelaar EF et al. The effect of host genetics on the gut microbiome. *Nat Genet* 2016;48:1407–12. 10.1038/ng.3663 27694959

[90] Wu G, Xu T, Zhao N et al. A core microbiome signature as an indicator of health. *Cell* 2024;187:6550–6565.e11. 10.1016/j.cell.2024.09.019 39378879

[91] Watson AR, Füssel J, Veseli I et al. Metabolic independence drives gut microbial colonization and resilience in health and disease. *Genome Biol* 2023;24:78. 10.1186/s13059-023-02924-x 37069665 PMC10108530

[92] Marcos S, Odriozola I, Aizpurua O et al. Functional gut microbiota dynamics of generalist and specialist bacteria in association with chicken growth. *ISME Commun* 2026;ycag091. 10.1093/ismeco/ycag091 42111291 PMC13155120

[93] Veseli IA, Chen YT, Schechter MS et al. Microbes with higher metabolic independence are enriched in human gut microbiomes under stress. *eLife* 2025;12:RP89862. 10.7554/eLife.89862 PMC12084026 40377187

[94] Wang J, Meier S, Soininen J et al. Regional and global elevational patterns of microbial species richness and evenness. *Ecography (Cop)* 2017;40:393–402. 10.1111/ecog.02216

[95] Lear G, Washington V, Neale M et al. The biogeography of stream bacteria: the biogeography of stream bacteria. *Glob Ecol Biogeogr* 2013;22:544–54. 10.1111/geb.12046

[96] Larkin AA, Martiny AC. Microdiversity shapes the traits, niche space, and biogeography of microbial taxa. *Environ Microbiol Rep* 2017;9:55–70. 10.1111/1758-2229.12523 28185400

[97] Montoya-Ciriaco N, Gómez-Acata S, Muñoz-Arenas LC et al. Dietary effects on gut microbiota of the mesquite lizard Sceloporus grammicus (Wiegmann, 1828) across different altitudes. *Microbiome* 2020;8:6. 10.1186/s40168-020-0783-6 31980039 PMC6982387

[98] Zhao F, Yang L, Zhang T et al. Gut microbiome signatures of extreme environment adaption in Tibetan pig. *NPJ Biofilms Microbiomes* 2023;9:27. 10.1038/s41522-023-00395-3 37225687 PMC10209067

[99] Ferrie GM, Alford VC, Atkinson J et al. Nutrition and health in amphibian husbandry: ex situ amphibian health and nutrition. *Zoo Biol* 2014;33:485–501. 10.1002/zoo.21180 25296396 PMC4685711

[100] Trochet A, Deluen M, Bertrand R et al. Body size increases with elevation in Pyrenean newts (Calotriton asper). *Herpetologica* 2019;75:30–7. 10.1655/D-18-00011

[101] Gaun N, Pietroni C, Martin-Bideguren G et al. The earth Hologenome initiative: data release 1. *Gigascience* 2025;14:giaf102. 10.1093/gigascience/giaf102 40910796 PMC12412122

